# Dysphagia in an 80-Year-Old Woman: A Rare Case of Metastatic Melanoma Presenting in the Small Bowel

**DOI:** 10.7759/cureus.71373

**Published:** 2024-10-13

**Authors:** Fatimah Zahra Rajabally, Jonathan Flynn, Faisal Abbasakoor, Shabir Ghanty

**Affiliations:** 1 School of Medicine, Royal College of Surgeons in Ireland, Dublin, IRL; 2 General Surgery, Clinic Darné Floréal C-Care, Floréal, MUS; 3 Radiology, Clinic Darné Floréal C-Care, Floréal, MUS

**Keywords:** dysphagia in elderly, jejunal mass, : metastatic melanoma, s: anemia, surgical laparotomy

## Abstract

This report discusses the case of an 80-year-old female presenting with non-specific gastrointestinal symptoms and who was ultimately diagnosed as having metastatic melanoma of the jejunum. Notably, on admission, the patient had failed to report a past medical history of melanoma on her back a few years prior, and this significantly impacted the diagnostic process. This case highlights the challenges in diagnosing metastatic melanoma. It adds to the literature by underscoring the rarity and diagnostic complexity of this condition, which in this particular case presented as a jejunal mass.

## Introduction

Metastatic melanoma is a malignant tumor originating from melanocytes, neural crest-derived cells found in the basal layer of the epidermis located in the skin, hair, uvea, mucosal epithelia, and meninges [1]. While melanoma is primarily detected in the skin, it can metastasize to various organs, including the lungs, liver, brain, bones, and gastrointestinal system. Gastrointestinal metastases, particularly to the small intestine, are rare, occurring in approximately 2% of melanoma patients [1]. Primary melanoma of the small intestine is exceedingly rare, with around 40 cases published in the current literature [2].

Melanoma incidence has been rising globally, with significant regional and ethnic variations. Countries with high incidence rates include New Zealand and parts of North America and Europe; however, the rates in Australia are particularly the highest globally [3]. Individuals with fair skin, a history of sunburn, excessive ultraviolet (UV) exposure, and a family history of melanoma are at higher risk [2,4]. According to the National Cancer Registry Report of Mauritius in 2022, 16 cases of melanoma were documented in the country (population around 1.2 million) [5]. Genetic predisposition plays a notable role in melanoma susceptibility, with approximately 5-10% of cases being familial [4]. The main oncogenic driver mutations in cutaneous malignant melanoma are the BRAF (serine/threonine protein kinase) and NRAS (neuroblastoma RAS viral oncogene) mutations [6]. Other susceptibility genes, such as CDKN2A, CDK4, and MITF, have been identified, although many familial cases lack established mutations in known genes [7].

The clinical presentation of gastrointestinal metastases from melanoma can be non-specific, including symptoms such as dyspepsia, epigastric pain, abdominal pain, nausea, vomiting, diarrhea, weight loss, and less commonly, complications like ulceration, perforation, and intussusception [8]. These symptoms can lead to incidental findings during surgeries or imaging for unrelated issues, complicating diagnosis [1].

The prognosis for patients with metastatic melanoma to the small bowel is generally poor, with median survival rates varying from six to 12 months after diagnosis [9]. However, early identification and surgical intervention can significantly improve outcomes. This case contributes to the limited literature on small bowel melanoma, emphasizing the need for heightened clinical suspicion and careful histopathological assessment in atypical presentations [10]. The rarity and aggressive nature of such malignancies underscore the challenges in diagnosis and management, as illustrated in this report.

## Case presentation

An 80-year-old Mauritian female of Caucasian origin was admitted to the hospital with complaints of worsening dysphagia, fatigue, and significant weight loss over the last couple of months. She had a history of well-controlled hypertension and diverticular disease. On admission, she did not mention any other significant medical history; she seemed in good condition, and her physical examination was unremarkable. Initial investigations revealed severe anemia (hemoglobin 7.0 g/dL, transferrin saturation 0.04 μmol/L), leukocytosis (12.20 ×109/l), and thrombocytosis (642,000 platelets/mm3).

**Figure 1:**
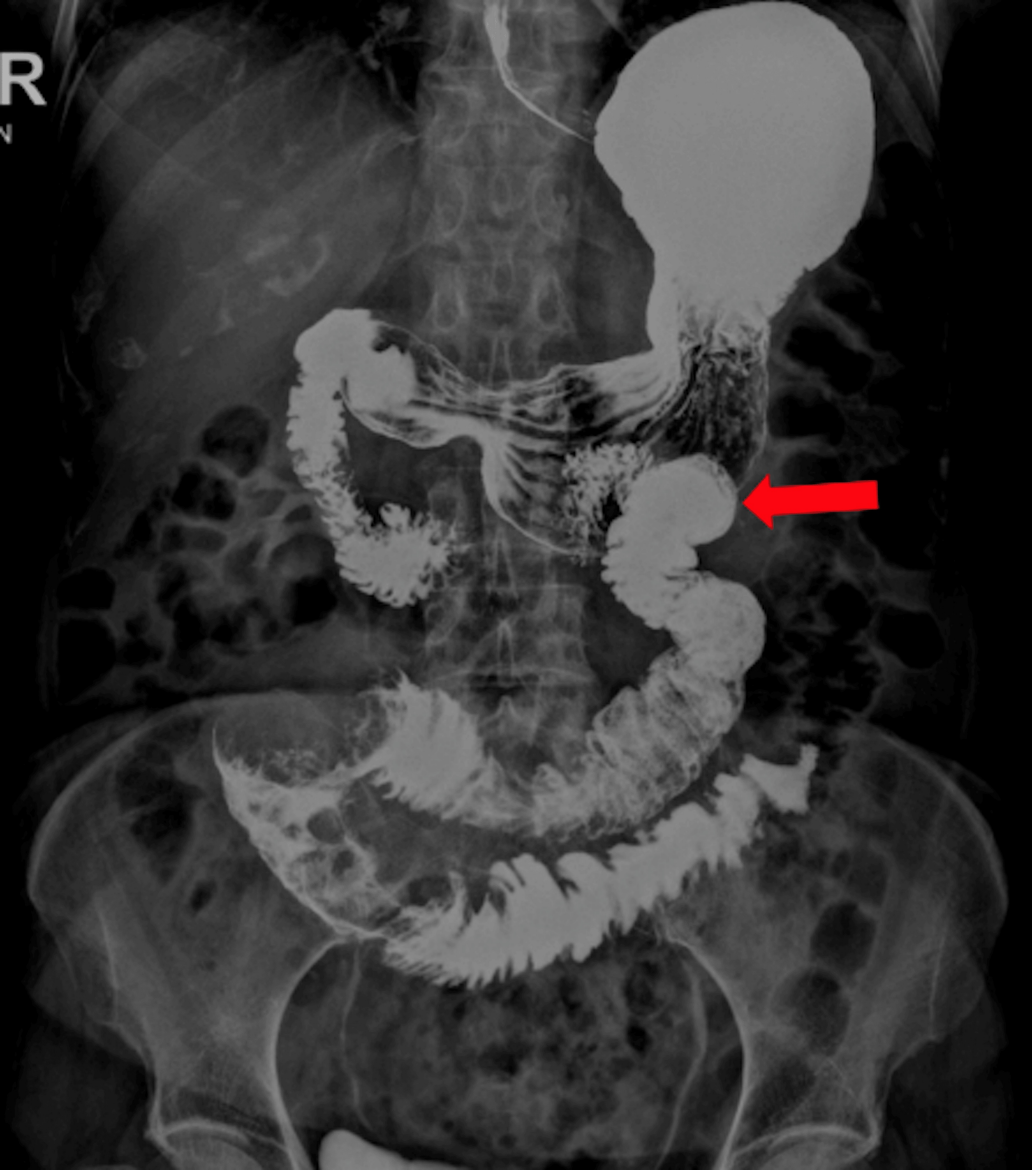
X-ray of the abdomen (anterior-posterior supine and erect views), barium meal, and follow-through showing filling defects of 5.8 cm and 5.2 cm seen at the proximal jejunal loop (red arrow).

**Figure 2:**
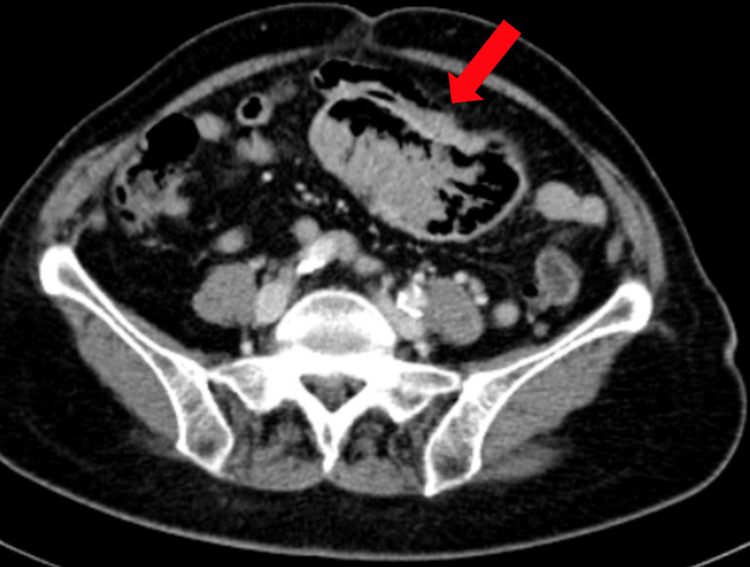
CT scan with oral-rectal water and IV contrast of the abdomen showing a small bowel mass lesion (red arrow) at the mid-abdomen.

Further evaluation was performed, including a plain abdomen X-ray and a barium meal revealing filling defects in the proximal jejunal loop. This raised suspicion of neoplastic lesions (Figure 1). Further imaging with a CT scan using oral-rectal water and IV contrast identified a large mass in the mid-left abdomen extending approximately 6.5 cm in the axial plane (Figure 2).

**Figure 3:**
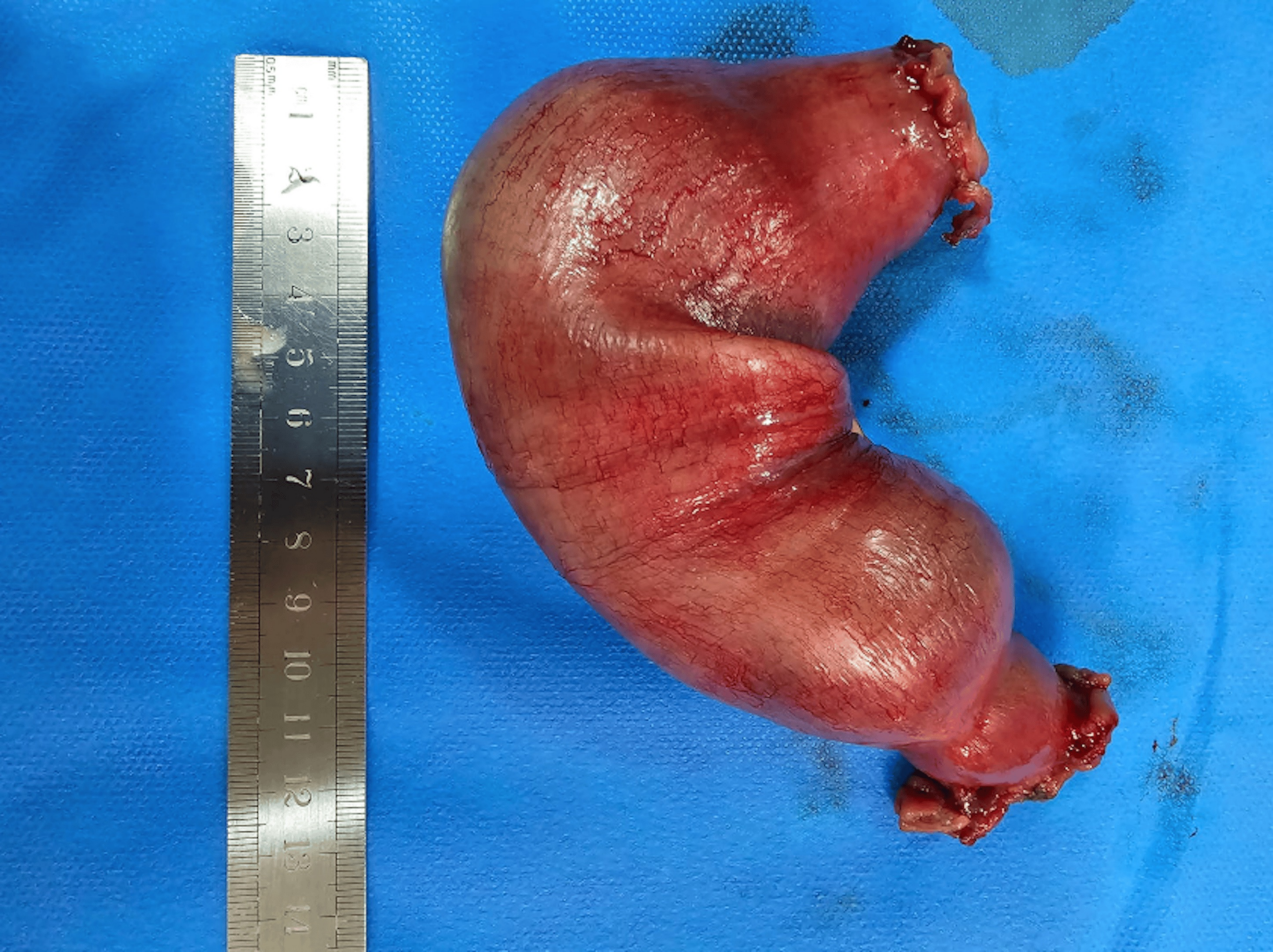
A segment of small intestine removed, 17 cm long. There is a sausage-shaped swelling centrally over approximately 10 cm.

The patient was referred to a colorectal surgeon, and during the laparotomy, a mass of approximately 15 cm was found in the small bowel, below the duodenojejunal flexure. A successful surgical segmental resection of the jejunal mass and anastomosis was performed. The excised tissue was then sent for histopathological examination (Figure 3). Postoperatively, she showed good and rapid recovery.

The histopathological analysis revealed “sheets of loosely cohesive polygonal cells with large vesicular nuclei and abundant brown pigment focally appearances suggest a malignant melanoma of the small intestine that has infiltrated through the muscularis propria but does not reach a peritonealized surface. No lymphovascular permeation is identified. The end resection margins are clear.”

An immunohistochemistry panel was performed to confirm the diagnosis. The tumor cells tested positive for melanoma markers, including S100, Melan-A, HMB45, and MIB1, confirming the melanocytic origin of the cells. The strong positivity for these markers is consistent with the diagnosis of metastatic melanoma.

The immediate prognosis for this case was cautiously optimistic given the absence of lymphovascular permeation and clear resection margins. However, due to the aggressive nature of metastatic melanoma, continued oncological surveillance and management are imperative to monitor for potential recurrence or further metastatic spread. She was scheduled for a follow-up review with an oncologist to discuss further management, including potential adjuvant therapy and long-term surveillance for metastatic melanoma.

## Discussion

Primary melanoma arises from melanocytes, generally in the skin, but can also develop in the eyes, mucous membranes, or other regions with melanocytes. It begins as a single tumor and is usually identified early by visual changes on the skin [11]. In contrast, secondary melanoma, or metastatic melanoma, occurs when the primary melanoma spreads to other organs, including the lymph nodes, liver, lungs, brain, and gastrointestinal tract [12]. This can be challenging to distinguish because metastatic melanomas often retain the same histological characteristics as the primary tumor. Without a known history of primary melanoma, secondary melanomas can be mistaken for primary tumors of the organ in which they appear [11]. This was evident in our case, where it was only after the patient mentioned previous surgery for melanoma removal on her back that we confirmed this was a secondary melanoma. This case underscores the critical importance of thorough patient history in accurately diagnosing and managing metastatic melanoma, as the patient's failure to report a previous melanoma significantly impacted the initial diagnostic process.

Epidemiological data from several studies show that small bowel involvement occurs in around 35%-70% of patients with known primary cutaneous metastatic melanoma [1,10,11]. Metastatic melanoma tends to extend to the small intestine (51-71%), notably to the jejunum and ileum [7]. This might be explained by the high expression of CCR9, a chemokine receptor, on melanoma cell surfaces. CCL25, the CCR9 ligand, is abundantly expressed in the small intestine [7]. Yet, less than 10% of metastatic lesions are discovered pre-mortem [1]. As a result, there are relatively few large-scale clinical investigations directly focused on metastatic melanoma involving the small bowel, and the literature on the subject is primarily made up of case reports, with few minor retrospective studies [1].

Moreover, this emphasizes the rarity of this condition, whereby metastatic disease may present with unusual clinical manifestations and can be misdiagnosed as primary tumors of the involved organs, further complicating the clinical picture and delaying appropriate treatment [12]. For instance, a study conducted by Bacchi et al. [13] highlighted metastatic melanoma presenting as an isolated breast tumor, clinically and morphologically resembling primary breast carcinoma. Another case by Mardi et al. [14] described metastatic melanoma appearing as a cystic mass in the thigh, initially thought to be a soft tissue sarcoma due to its unusual presentation. This underscores the need for clinicians to maintain a high index of suspicion and perform comprehensive evaluations when assessing patients with atypical presentations or a history of melanoma.

Metastatic melanoma diagnosis requires a variety of imaging and endoscopic procedures to identify and diagnose the progression of the disease effectively. Abdominal ultrasonography is generally ineffective in recognizing gastrointestinal malignant melanomas [7,15]. Barium evaluations are common procedures for locating intestinal melanoma, although they do not reveal extraintestinal abnormalities [7,15]. Cross-sectional CT imaging has a 60-70% sensitivity for revealing intestinal melanoma metastases, while CT enteroclysis has increased detection rates [15,16]. Whole-body PET-CT had higher sensitivity and specificity than CT for detecting all gastrointestinal metastatic melanomas [16]. Endoscopic procedures such as upper and lower gastrointestinal (GI) endoscopy, video capsule endoscopy, and enteroscopy also aid in diagnosis [7,15]. These recent advancements in imaging techniques have strengthened diagnostic accuracy, decreased unnecessary biopsies, and improved treatment plans for metastatic melanoma [16]. In our patient’s case, the pre-operative barium meal and CT scan indicated a possible neoplasm. Still, it was only immunohistochemical findings and the patient’s late revelation of her history of back melanoma that confirmed metastatic melanoma.

The treatment options for metastatic melanoma include surgery and systemic treatments, which are customized to the individual patient based on the location and number of metastases. Surgery is commonly performed to remove solitary metastases, especially when tumors produce symptoms or are easily accessible. In the case of our patient, surgery was the best option since she was in good clinical condition and the small bowel metastasis was fully resectable. Studies have shown that surgical treatment for patients with small bowel metastases from melanoma can significantly increase survival rates. Specifically, the five- and 10-year survival rates for patients who underwent curative-intent resection were 61% and 54%, respectively, compared to just 4% for those who received palliative surgery [17,18,19]. Furthermore, the absence of any residual disease after surgery was associated with better survival outcomes, regardless of metastases in other organs [18]. Surgical resection of the affected bowel segment also provided immediate relief of these symptoms and improved the patient’s quality of life.

Systemic treatments like immunotherapy are essential as they build the body’s immune response against cancer cells with the help of checkpoint inhibitors. These inhibitors help the immune system recognize and attack cancer cells by “taking the brakes off” immune cells [20].

Pembrolizumab, a checkpoint inhibitor targeting the PD-1 pathway, helps the immune system recognize and destroy melanoma cells more effectively [20]. According to Knight et al., pembrolizumab significantly improves survival rates for patients with advanced melanoma, reduces the risk of recurrence, and improves overall survival rates in patients with resected melanoma [20].

Targeted therapy for melanoma involves using drugs that specifically target genetic mutations and proteins needed for melanoma cell growth and survival, making treatment more effective and less harmful to normal cells [21]. About 50% of melanomas have mutations in the BRAF gene, leading to uncontrolled cell growth [21]. BRAF inhibitors like vemurafenib and dabrafenib target this mutation, inhibiting tumor growth [22,23]. These inhibitors are often used with MEK inhibitors (e.g., trametinib), providing a more comprehensive approach to blocking cancer cell proliferation and survival. According to Aya et al. [23], simultaneously targeting BRAF mutations and MEK significantly improves treatment outcomes in patients with metastatic melanoma, with prolonged progression-free survival and overall survival compared to monotherapy.

## Conclusions

This case report illustrates the diagnostic challenges of metastatic melanoma presenting with non-specific gastrointestinal symptoms. The patient’s failure to report a previous melanoma history initially complicated the diagnostic process. Surgical resection and histopathological confirmation were critical in managing this case, emphasizing the importance of multidisciplinary teams combining clinical, radiological, and pathological assessments. Continuous oncological surveillance remains essential due to the aggressive nature of metastatic melanoma.
